# A Novel Real-Time PCR Assay for the Rapid Detection of Virulent *Streptococcus equi* Subspecies *zooepidemicus*—An Emerging Pathogen of Swine

**DOI:** 10.3389/fvets.2021.604675

**Published:** 2021-02-12

**Authors:** Suresh V. Kuchipudi, Meera Surendran Nair, Michele Yon, Abhinay Gontu, Ruth H. Nissly, Rhiannon Barry, Denver Greenawalt, Traci Pierre, Lingling Li, Nagaraja Thirumalapura, Deepanker Tewari, Bhushan Jayarao

**Affiliations:** ^1^Animal Diagnostic Laboratory, Pennsylvania State University, Wiley Lane, University Park, PA, United States; ^2^Center for Infectious Disease Dynamics, Pennsylvania State University, University Park, PA, United States; ^3^Pennsylvania Veterinary Laboratory, Harrisburg, PA, United States

**Keywords:** *SzM* gene, real time PCR, pig mortality, virulent strains, *Streptococcus equi* subspecies *zooepidemicus*

## Abstract

*Streptococcus equi* subspecies *zooepidemicus*, a zoonotic bacterial pathogen caused a series of outbreaks with high mortality affecting swine herds in multiple locations of the USA and Canada in 2019. Further genetic analysis revealed that this agent clustered with ATCC 35246, a *S. zooepidemicus* strain associated with high mortality outbreaks in swine herds of China originally reported in 1977. Rapid and accurate diagnosis is absolutely critical for controlling and limiting further spread of this emerging disease of swine. Currently available diagnostic methods including bacteriological examination and PCR assays do not distinguish between the virulent strains and avirulent commensal strains of *S. zooepidemicus*, which is critical given that this pathogen is a normal inhabitant of the swine respiratory tract. Based on comparative analyses of whole genome sequences of the virulent isolates and avirulent sequences, we identified a region in the *SzM gene* that is highly conserved and restricted to virulent *S. zooepidemicus* strains. We developed and validated a novel probe-based real-time PCR targeting the conserved region of *SzM*. The assay was highly sensitive and specific to the virulent swine isolates of *Streptococcus equi* subspecies *zooepidemicus*. No cross reactivity was observed with avirulent *S. zooepidemicus* isolates as well as other streptococcal species and a panel of porcine respiratory bacterial and viral pathogens. The PCR efficiency of the assay was 96.64 % and was able to detect as little as 20 fg of the bacterial DNA. We then validated the diagnostic sensitivity and specificity of the new PCR assay using a panel of clinical samples (*n* = 57) and found that the assay has 100% sensitivity and specificity as compared to bacteriological culture method. In summary, the PCR assay will be an extremely valuable tool for the rapid accurate detection of virulent swine *S. zooepidemicus* isolates and directly from clinical samples.

## Introduction

*Streptococcus equi* subspecies *zooepidemicus* (referred as *S. zooepidemicus* hereafter), is a zoonotic pathogen of importance to animal and human health and is often associated with sudden epizootics in animals and hence the name *zooepidemicus* (1). *S. zooepidemicus* is known to be an opportunistic pathogen affecting a wide range of hosts including horses, pigs, ruminants, guinea pigs, monkeys, cats, dogs, poultry and humans (2–5). *S. zooepidemicus* infection is manifested in different forms in various hosts, and the disease symptoms include septicemia and arthritis in pigs; mastitis, arthritis, respiratory, and uterine infections in horses; metritis and mastitis in cattle; glomerulonephritis, rheumatic fever, meningitis and purulent arthritis in humans (3, 6, 7). *S. zooepidemicus* is known to cause pneumonia in equines and canines (5, 8).

Outbreaks of *S. zooepidemicus* infection in pigs and monkeys with significant morbidity and mortality have been reported previously in Asia (9, 10) and recently in North America (11). *S. zooepidemicus* infections resulting in sudden death and abortions with high mortality in commercial swine farms were reported in 2019 from the province of Manitoba in Canada (12). Subsequently, several outbreaks of *S. zooepidemicus* in commercial swine farms with high morbidity and mortality have been reported from Ohio, Tennessee and Pennsylvania in the US (11, 13). The mortality in these outbreaks ranged from 10 to 50% and characterized by sudden onset of death, weakness, lethargy, hyperthermia, and post-mortem lesions included splenomegaly and hemorrhagic lymph nodes. An investigation into the recent outbreaks revealed a striking homology between the recent outbreak isolates and strains of *S. zooepidemicus* that caused outbreaks with high mortality in swine populations of China in 1977 and with three isolates from human cases with guinea pig exposure (13). Further, the genome analysis of these North American isolates indicated that the virulent swine strains of *S. zooepidemicus* are epidemiologically related and were significantly different from other swine isolates and most isolates from other animal species (13).

Diagnosis of streptococcal infections is traditionally based on clinical signs and pathological findings in conjunction with laboratory isolation and biochemical identification of the causative agent. However, accurate laboratory identification of *S. zooepidemicus* poses a challenge as it shares >98% homology in the DNA sequence to the other subspecies, including *S. equi* subspecies *equi* (referred as *S. equi* hereafter), the etiological agent of strangles in horses (6). Molecular tests such as PCR targeting the bacterial genome and mass spectrometric analysis of ribosomal proteins from isolated bacterial colonies have been used in recent years to aid rapid detection and differentiation of *S. equi* subspecies (14, 15). A dual-target PCR is routinely used in many laboratories to differentiate *S. equi* subspecies- *S. equi* and *S*. *zooepidemicus* (14). However, all these methods cannot distinguish virulent and avirulent strains of *S. zooepidemicus* and will not accurately differentiate a mixed culture of *S. equi* subspecies- *S*. equi and *S. zooepidemicus*. It is important to note that mere detection of *S*. *zooepidemicus* from animal specimens is of little clinical value as these organisms are found as commensals in many animals. Currently there are no molecular diagnostic assays that can differentiate the virulent *S. zooepidemicus* isolates responsible for causing lethal disease outbreaks from the rest of the avirulent strains *S. zooepidemicus*. Therefore, sensitive and specific methods to selectively identify the virulent *S. zooepidemicus* isolates is urgently needed for early diagnosis and outbreak control.

Several genomic islands and virulence genes in the *S. zooepidemicus* isolates were attributed to the high mortality in swine populations. *SzM* gene, which encodes an M-like protein and fibrinogen binding properties, was identified through comparative genome analysis as a key virulence factor of *S. zooepidemicus* for swine (13). *SzM* gene is a partial analog of a major virulence factor, *SeM* of *S. equi* (16). *SzM* is highly conserved with 100% homology in the virulent swine isolates including ATCC 35246, and not present in the avirulent *S. zooepidemicus* strains (13). Similar findings have also been observed with the lethal outbreak isolates from Pennsylvania (PA) and the homology findings are discussed here along with the results to the PCR development and validation experiments described. Therefore, in light of previous genome analysis reports and our findings with the PA isolates, *SzM* was selected as the target for the development of a probe-based real-time PCR diagnostic assay for the detection of virulent *S. zooepidemicus* isolates.

## Materials and Methods

### Bacterial Isolates

Two bacterial cultures were isolated in December 2019 in Pennsylvania from a swine herd, which experienced high mortality. The pure cultures were confirmed as *S. zooepidemicus* initially using matrix assisted laser desorption ionization-time of flight mass spectrometry (MALDI-TOF MS) followed by whole genome sequencing. The raw reads have been submitted to the SRA database under the BioProject accession number PRJNA591128. The annotated full genomes of the isolates have been deposited in GenBank under the accession numbers JABDID000000000 and JABMIH000000000.

### Development of the qPCR Assay

Comparative gene-based analysis of the two isolates was performed along with virulent type strain ATCC 35246 using Molecular Evolutionary Genetics Analysis software (MEGA-X®) (17). The gene *SzM* was conserved among the two isolates and was earlier reported to be present only in the virulent strains of *S. zooepidemicus* from swine (13). Primers and probe targeting 85 bp region of the *SzM* gene were designed using Primer Express v. 3.0.1® (Applied Biosystems). NCBI Primer-Blast® analysis was used to confirm the specificity of primers by confirming the absence of targets other than virulent strains of *S. zooepidemicus* in the nucleotide sequence database (18). The sequence of *SzM* in *S. zooepidemicus* isolates from other animals for example equines is different and hence this assay will not cross react and is specific for virulent swine isolates. The forward primer (5′—AAGTCGTTGCTCAACTTCATCTATTAAC–3′), reverse primer (5′—TAGGTAATGACCGTCCTAATGATGTT–3′) and the probe (5′—6FAM-AGTTTAACCCTCTTGATCTAT-MGBNFQ–3′) were manufactured by Applied Biosystems, USA. The qPCR assay mixture consisted of 0.4 mM of each primer, 0.3 mM probe and VetMax-Plus qPCR Master Mix® (Applied Biosystems, USA) for a volume of 20 μl. A template DNA volume of 5 μl was added and the assay was performed on an ABI 7500 FAST system® (Applied Biosystems, USA). The optimal cycling conditions were standardized as: 95°C for 10 min followed by 40 cycles of 95°C for 15 s and 60°C for 45 s, with data collection at the end of each 60°C step.

### Validation of the qPCR Assay

Assay specificity was tested with a panel of reference bacterial isolates and viral pathogens commonly associated with porcine respiratory disease syndrome (Table 1). Additionally, commensal strains of *S. zooepidemicus* isolated from horses (*n* = 10) obtained from Pennsylvania were tested by the developed assay. Avirulent *S. zooepidemicus* isolates obtained from diagnostic cases submitted to the Pennsylvania Animal Diagnostic Lab system (PADLS), were also tested with the developed assay. All the isolates were confirmed by MALDI-TOF MS and conventional bacterial identification (data not shown).

**Table 1:** Panel of microbial pathogens used for validating the specificity of the developed PCR assay.

**Specimen**	**Reference number**	**Specimen**	**Reference number**
*Streptococcus equi* subspecies *equi*	ATCC 33398	*Streptococcus equi* subspecies *zooepidemicus (avirulent)* (commensal isolates fr)	PADLS S2003641
*Streptococcus equi* subspecies *zooepidemicus*	ATCC 43079		PADLS S2008462
*Streptococcus agalactiae*	ATCC 12386		PADLS N2007902
*Streptococcus pneumoniae*	ATCC 49619		PADLS N2002258
*Group C Streptococcus*	ATCC 12449, ATCC 9892		PADLS N2010811
*Actinobacillus pleuropneumoniae*	ATCC 27088		PADLS N2010748
*Mycoplasma hyopneumoniae*	ATCC 25095		PADLS N2002716
*Pasteurella multocida*	ATCC 9659		PADLS N2008343
*Porcine Circovirus II*	American Bioresearch Laboratories 240-53		PADLS N2008632
*Porcine Pseudorabies Virus*	NVSL 070PDV		PADLS N2003366
Encephalomyocarditis Virus^*^	ATCC VR-1762	Porcine Reproductive and Respiratory Syndrome Virus^*^	NVSL 130PDV
Porcine Respiratory Coronavirus^*^	ATCC VR-2384	Swine Influenza Virus^*^	NVSL 003IDV

*ATCC, American Type Culture Collection; NVSL, National Veterinary Services Laboratory; PADLS, Pennsylvania Animal Diagnostic Laboratory System*.

* *RNA viruses associated with porcine respiratory disease complex*.

The analytical sensitivity and limit of detection was determined using serial dilutions of the *S. zooepidemicus* DNA. The diagnostic sensitivity and specificity of the PCR assay was established comparing with bacterial culture method using samples (*n* = 57) from pigs with and without respiratory disease that were submitted to the Animal Diagnostic Lab. The PCR assay was also tested for its range of detection, linearity, efficiency, precision, and repeatability. The validation of the assay was performed based on the guidelines laid out by American Association of Veterinary Laboratory Diagnosticians (AAVLD).

## Results

### Assay Specificity

The developed qPCR failed to amplify any region in the tested related and unrelated pathogens which can cause porcine respiratory diseases. The panel of pathogens also included avirulent *S. zooepidemicus*, which was not amplified by the assay.

### Analytical Sensitivity, Limit of Detection, and Efficiency

**Figure 1:**
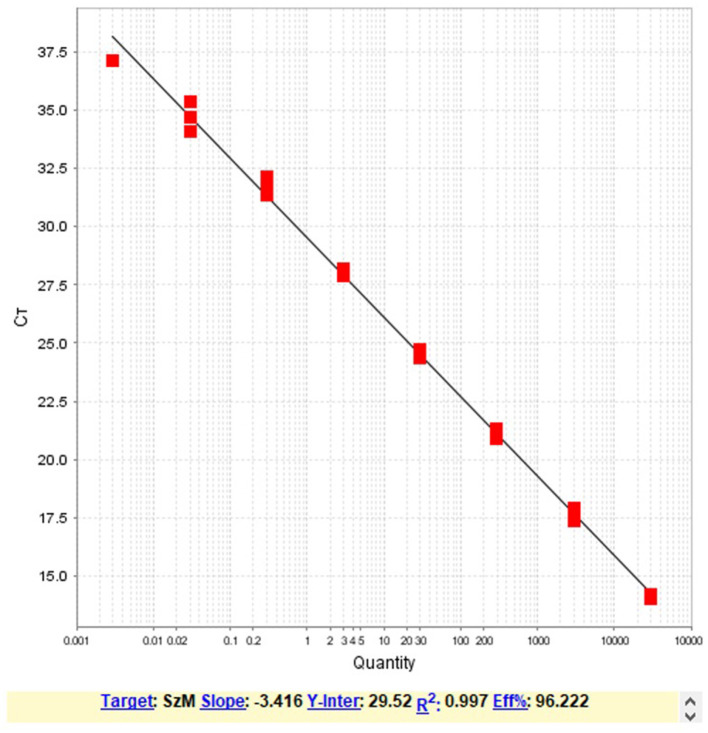
The regression analysis of the resultant average Ct value with the Log_10_ dilutions of the DNA template showing the linearity and the efficiency of the PCR assay.

Triplicates of ten-fold dilutions of the DNA from one of the virulent *S. zooepidemicus* were tested by the developed assay. The linearity (Figure 1), range of detection and efficiency of the assay was determined (Table 2). The analytical sensitivity corresponding to the lowest limit of detection was determined as 20 fg of the target DNA (Ct = 34.10 ± 0.61).

**Table 2:** The Ct values for the log dilutions of the DNA template, giving the range of maximal and minimal dilution of CFU of the pathogen which could be detected by the developed PCR assay.

**Dilution**	**Replicate-1**	**Replicate-2**	**Replicate-3**	**CV%**
Undiluted	14.04	14.19	13.99	0.71
1x10^−1^	17.93	17.5	17.37	1.67
1x10^−2^	21.29	21.1	20.88	0.96
1x10^−3^	24.69	24.52	24.34	0.70
1x10^−4^	28.16	28.06	27.87	0.54
1x10^−5^	32.16	31.68	31.33	1.31
1x10^−6^	34.65	35.33	34.1	1.79
1x10^−7^	ND	ND	37.1	ND

*CFU, Colony forming unit; ND, Not determined; CV, Coefficient of variance*.

### Precision and Repeatability

The resulting Ct values from five replicates of three dilutions of the template DNA tested within a day was analyzed to calculate the Coefficient of variance (CV) % ranging from 0.19 to 1.16 % (Table 3). The inter-day variation of the assay ranged between a CV % of 0.51 to 1.49 % (for dilutions from 10^∧^−1 to 10^∧^−3) (Table 3). The intermediate precision both within day and between days resulting in <2 % CV, confirmed high repeatability of the assay.

**Table 3:** Intra-day and inter-day precision of the resulting Ct values from five replicates tested each day for eight days were <2 % co-efficient of variance.

**Dilution of template DNA**	**Intra-day precision (CV %)**								**Inter-day precision (CV)**
	**Day 1**	**Day 2**	**Day 3**	**Day 4**	**Day 5**	**Day 6**	**Day 7**	**Day 8**	
1 x 10^−1^	0.19	0.20	0.58	0.51	0.27	0.23	0.57	1.15	1.49
1 x 10^−2^	0.21	0.23	1.11	0.42	0.52	0.59	0.59	0.78	0.84
1 x 10^−3^	0.21	0.50	0.82	1.03	0.77	1.16	0.40	0.36	0.51

*CV, Coefficient of variance*.

### Diagnostic Sensitivity and Specificity

We used a panel of porcine clinical samples that comprised tissues, contact swabs or isolated cultures that were examined by culture followed by MALDI-TOF MS identification. Both the PCR and culture method identified 27 samples as positive and 30 samples as negative, confirming the 100% sensitivity and specificity of the PCR assay. In addition, we extracted DNA from avirulent *S. zooepidemicus* isolates which gave a negative test as expected.

## Discussion

Evolution of microbial pathogens is an ongoing process, and new pathogens are continually emerging from nature. In addition to the newly emerging pathogens, reemergence of pathogens into new regions continues to be a major global threat to animal and human health. Emerging and remerging animal infectious diseases have the potential to negatively impact animal health, food safety and trade. In addition, several animal infectious diseases have zoonotic potential and hence can have a significant impact on public health.

The recent outbreaks of a virulent S. *zooepidemicus* flag the re-emergence of the infection after more than four decades of its appearance in China in the 1970s. Conventionally, differential diagnosis of *S. zooepidemicus* from the closely related *S. equi* subspecies often involve biochemical characterization, mass spectrometric analysis, polymerase chain reactions or genome sequence analyses (14, 19, 20). The molecular diagnostics described to date are largely focused on presence of genes of *S. equi* and their corresponding absence in *S. zooepidemicus*. Båverud et al. (14) described a PCR method which amplifies regions in *SodA* and *SeeI* genes (14). The superoxide dismutase A (*SodA*) gene is amplified in both the subspecies- *S. equi* and *S. zooepidemicus*, which were later differentiated by amplification of *SeeI* in *S. equi* isolates alone. A few diagnostics were based on detection of *SeM*, an M-like protein of *S. equi* as a unique gene absent in *S. zooepidemicus* (6, 21). *SeeH* and *SeeI* genes were also targeted with the purpose of identifying *S. equi* from *S. zooepidemicus* (22). All the above-mentioned molecular diagnostics have limitations in that they cannot distinguish a mixed cultures of *S. equi* and *S. zooepidemicus*. Moreover, all the described two-step PCR assays are based on the absence of *SeeI* genes for confirming the presence of *S. zooepidemicus*. As such these assays are useful to establish that *S. zooepidemicus* is not present (rule out) but are not very specific to confirm (rule in) the diagnosis. Furthermore, the analyses of 16s rRNA reveal that the sequences are identical among the *S. equi* strains but vary widely among the *S. zooepidemicus* strains (14). There is a wide genetic variation reported among isolates of *S. zooepidemicus* as compared to *S. equi* subspecies (23).

With the recent outbreak of *S. zooepidemicus*, the swine farms have been kept on high alert owing to the high mortality produced by the pathogen. Accurate and rapid diagnosis of the virulent *S. zooepidemicus* is of utmost importance to not only treat the affected animals but also to swiftly implement mitigation measures to further prevent the spread of this deadly infection. The genome sequencing analyses of the isolates from North America revealed the various virulence factors and their conserved nature among the virulent strains of *S. zooepidemicus- SzM, lmb, fbpz, skc, has* operon & *mag* regulon (12). Our genomic analyses of the isolates from the recent US outbreak with other virulent strains of *S. zooepidemicus* revealed that *SzM*, the gene for a M-like protein of the bacterium is highly conserved and an important virulence contributing factor as was previously reported (13).

The probe based real-time PCR assay developed and validated in this study provides a highly specific means to make a rule in diagnosis of the virulent S. *zooepidemicus* infection. As this assay can differentiate the virulent *S. zooepidemicus* isolates from both avirulent *S. zooepidemicus* and *S. equi* isolates, it could help in better understanding the ecology and epidemiology of these bacterial agents. A key question yet to be answered is whether susceptible animals of other species serve as reservoirs for *S. zooepidemicus*. This PCR assay can be used to answer this question and further investigate the host range *S. zooepidemicus*. In summary, this novel assay which can give the result in <4 h provided a practical solution to the hitherto unsolved problem of diagnosing virulent *S. zooepidemicus*.

## Data Availability Statement

The raw reads have been submitted to the SRA database under the BioProject accession number PRJNA591128. The annotated full genomes of the isolates have been deposited in GenBank under the accession numbers JABDID000000000 and JABMIH000000000.

## Author Contributions

SK conceived the study. MY and MS assisted in study design. RN, RB, DG, TP, LL, NT, DT, and BJ helped in data collection, analysis, and interpretation. AG, MS, MY, and SK wrote the manuscript. All authors reviewed and approved the manuscript.

## Conflict of Interest

The authors declare that the research was conducted in the absence of any commercial or financial relationships that could be construed as a potential conflict of interest.
